# c.*84G>A Mutation in *CETP* Is Associated with Coronary Artery Disease in South Indians

**DOI:** 10.1371/journal.pone.0164151

**Published:** 2016-10-21

**Authors:** Mala Ganesan, Sheikh Nizamuddin, Shiva Krishna Katkam, Konda Kumaraswami, Uday Kumar Hosad, Limmy Loret Lobo, Vijay Kumar Kutala, Kumarasamy Thangaraj

**Affiliations:** 1 CSIR-Centre for Cellular and Molecular Biology, Hyderabad, India; 2 Department of Clinical Pharmacology and Therapeutics, Nizam's Institute of Medical Sciences (NIMS), Hyderabad, India; 3 Yashoda Super Specialty Hospital, Hyderabad, India; Kunming Institute of Zoology, Chinese Academy of Sciences, CHINA

## Abstract

**Background:**

Coronary artery disease (CAD) is one of the leading causes of mortality worldwide. It is a multi-factorial disease and several studies have demonstrated that the genetic factors play a major role in CAD. Although variations in cholesteryl ester transfer protein (*CETP*) gene are reported to be associated with CAD, this gene has not been studied in South Indian populations. Hence we evaluated the *CETP* gene variations in CAD patients of South Indian origin.

**Methods:**

We sequenced all the exons, exon-intron boundaries and UTRs of *CETP* in 323 CAD patients along with 300 ethnically and age matched controls. Variations observed in *CETP* were subjected to various statistical analyses.

**Results and Discussion:**

Our analysis revealed a total of 13 variations. Of these, one3’UTRvariant rs1801706 (c.*84G>A) was significantly associated with CAD (genotype association test: OR = 2.16, 95% CI: 1.50–3.10, p = 1.88x10^-5^ and allelic association test: OR = 1.92, 95% CI: 1.40–2.63, p = 2.57x10^-5^). Mutant allele “A” was observed to influence the higher concentration of mRNA (p = 7.09×10^−3^, R^2^ = 0.029 and β = 0.2163). Since expression of *CETP* has been shown to be positively correlated with the risk of CAD, higher frequency of “A” allele (patients: 22.69% *vs*.controls: 13%) reveals that c.*84G>A is a risk factor for CAD in South Indians.

**Conclusions:**

This is the first report of the *CETP* gene among South Indians CAD patients. Our results suggest that rs1801706 (c.*84G>A) is a risk factor for CAD in South Indian population.

## Introduction

Coronary artery disease (CAD) is the leading cause of mortality worldwide [1]. CAD and its clinical manifestations are etiologically complex, with approximately equal contributions from genetic and environmental factors [2,3]. Genetic risk scores derived from several functionally relevant single nucleotide polymorphisms (SNPs) or haplotypes in several genes may help in predicting CAD [4]. Associations of only a few common SNPs with CAD have been consistently replicated in several studies [5]. Both genome-wide association studies and candidate-gene approaches have identified a number of novel chromosome loci or genes that are associated with CAD [6,7]. However, they account for relatively small portion of the overall CAD risk; therefore, there is a need for identification of novel loci or genes for CAD. Cholesteryl ester transfer protein (*CETP*) gene is localized on chromosome 16, which is of 21995 base pairs (bp) in size and consists of 16 exons (Gene ID 1071).CETP is a hydrophobic glycoprotein which plays a major role in RCT (Reverse Cholesterol Transport) from tissues to the liver. By enabling transfer of cholesteryl esters from high-density lipoproteins (HDL) to low density lipoproteins (LDL) and very low density lipoproteins (VLDL) lipoproteins *CETP* enables remodeling of plasma lipoproteins [8]. The elevated level of HDL cholesterol (HDL-C) is shown to be a protective factor for coronary artery disease from many epidemiological studies [9].Our earlier studies have suggest that Indian populations are unique in their origin and practicing endogamy for the past thousands of years, hence expected to have unique set of mutation which led to several disease, cardiac disease in particular [10–12].

Among the fastest growing non-communicable diseases, cardiovascular diseases (CVDs) are expected to cause largest number of mortality and morbidity within India [13]. Indians possess a unique lipid profile characterized by high triglycerides, low high-density lipoprotein (HDL), and increased lipoprotein (a) levels [14]. *CETP* plays a major role in HDL metabolism and this gene possesses several SNPs that have been reported to be associated with plasma HDL concentrations. However, this gene has not been analysed on Indian population, hence this study was aimed to investigate whether *CETP* gene variations influence CAD in South Indian population.

## Materials and Methods

### 2.1. Sample details

The study subjects composed of 323 CAD patients with coronary atherosclerosis and 300 age and ethnically matched healthy controls from South India. Blood samples were collected from Yashoda Hospital and Nizam Institute of Medical Sciences (NIMS), Hyderabad, India. Clinical manifestation of coronary atherosclerosis was evaluated by precutaneous coronary angiography, by a panel of experienced cardiologists. All ethnically matched control individuals were free from CAD, as determined by medical history, clinical examinations, or electrocardiography. Among CAD patients, 70% were males and 30% were females, while among the healthy control group 76% were male and 24% were female. The mean age of healthy controls was 65.26±10.30 years while that of patients was 56.21±10.45 years. In CAD patients; 18.57% were tobacco chewers, while it was 10% among the healthy controls. The demographic details of the individuals included in the study are given in **Table 1**.

**Table 1:** Clinical detail of individuals included in the study.

Clinical details	Controls	CAD patients	P-value (test)
Number of samples	300	323	-
Linguistic affiliation	Dravidian (Telugu)	Dravidian (Telugu)	-
Sex (M:F)	228(76%):72(24%)	226(70%):97(30%)	0.1045 (chisq)
Age (years) at sampling (mean±S.D.)	65.26±10.30	56.21±10.45	2.36×10^−25^ (t)
Tobacco (Y/N/%)	30(10%) / 270(90%)	60(18.57%) /263(81.42%)	0.0029 (chisq)
Alcohol (Y/N) (N/%)	20(6.66%) / 280(93.33%)	43(13.31%) / 280(86.68%)	0.0075 (chisq)
Hypertension (Y/N) (N/%)	NA	180(55.72%)/55(44.27%)	-
Family history(Y/N) (N/%)	Nil	170(52.63%) /153(47.36%)	-
Total cholesterol (mg/dl) (mean±S.D.)	150.5±30.8	212.5±40.8	2.36×10^−76^ (t)
Triglycerides (mg/dl) (mean±S.D.)	100.2±50.5	150.2±100.5	1.55×10^−14^ (t)
HDL (mg/dl) (mean±S.D.)	52.2±10.1	39.2±20.9	1.86×10^−21^ (t)
LDL (mg/dl) (mean±S.D.)	88.8±28.4	98.8±50.4	0.0022 (t)
VLDL (mean±S.D.)	16.2±13.4	30.6±15.4	5.20×10^−32^ (t)

M- Male; F- Female; S.D.-Standard deviation Y—yes; N—no; mg/dl milligram/deciliter; chisq–Chi-square test; t–T-test

We also utilized the genotype data of rs1801706/c.*84G>A of 1000 genome project’s samples from Ensembl genome browser (version 84) (asia.ensembl.org).

### 2.2. Sample collection and DNA isolation

Prior to collection of blood samples, CAD patients were subjected to physical/clinical examinations such as 12 lead ECG and lipid profile, etc. Blood samples of healthy controls from the same ethnic background, without hypertension or CAD based on electrocardiograph were collected. A total of 10 ml intravenous blood samples of both cases and controls were collected in EDTA vaccutainer, after obtaining informed written consent. Genomic DNA was isolated from all the samples using standard protocol [15]. This study followed the principles outlined in the Declaration of Helsinki (WMA World Medical Association Declaration of Helsinki), and was approved by the Institutional Ethics Committee of Yashoda Hospital, Hyderabad; Nizam’s Institute of Medical Sciences (NIMS), Hyderabad; and CSIR-Centre for Cellular and Molecular Biology (CCMB), Hyderabad, India.

### 2.3. Genotyping

The reference genomic sequence of *CETP* (ENSG00000087237) was obtained from the Ensemble database (asia.ensembl.org). Primers to amplify all the exons, and exon-intron boundaries of *CETP* were designed using Primer3 web version 4.0 (**Table 2**). PCRs (polymerase chain reactions) were performed using GeneAmp 9700 (Applied Biosystems, Foster City, USA) using Emerald Amp GT PCR master mix (TaKaRa) according to the manufacturer's protocol. After PCR, Amplicons were size fractionated using2% agarose gel, stained with ethidium bromide and observed under UV transilluminator. Subsequently, amplicons were treated with Exo-SAP (USB Corp., USA), and sequenced using a BigDye Terminator (v3.1) cycle sequencing kit (Applied Biosystems, Foster City, USA) on an ABI 3730XL DNA analyzer. Sequences obtained were assembled with the reference sequences using AutoAssembler software (Applied Biosystems, Foster City, USA). Variations observed were noted for further analysis.

**Table 2:** Sequence of primers used to amplify exons and exon-intron boundaries of *CETP* gene. Annealing temperature of all the 12 set of primers are 60°C.

Exon #	Forward Primer	Reverse Primer
1	TGCCCGGAAGAGCCTCATGTT	CTCTTCCAGGATCGACGTAAC
2	CACTGCCCTCCCCTCTAG	GACCCCCATCCCTCCGCC
3	CTTCCACCCTCGCCTAGACAA	AAGGACGAGCACGGGTAGGAC
4	CATGGATGCACAGGACTGGTC	GTCTTCTGTCGTCACCCTCGG
5	GCGGTGACTCAGGGCAATTC	TCAACCAGGAAAAAACACGA
6	TCCCAATCTCCCTGAAGCTG	TGTGCGTACCCCTCCTCCCT
7	TGCCCTTGGTCCCTGCGA	GTGTCGAGGATGAGCCAA
8	CCCGGAGCCAGCTTTGTC	CTCCACCACCACCCCCTT
9	TCCCGTGTCATCCTTGCC	CAGTCCGTGTCCCGCCCC
10	GGAGGGCTGCCAGGAAGAAGG	ACTGGGAAGAAGAGGGACGGT
11	TGGGGCAGGAAAACGGAGTG	ATGAATCGCCAGGACCGGGG
12	GGTCCAAAAGGGTCTCAGCA	GCAGGTGGAGAAAAGTCGGG

### 2.4. Statistical analysis and functional validation

Allele and genotype frequencies were calculated by the allele counting method. Statistical comparisons were carried out by Plink software [16].The P values less than 0.05 were considered for statistical significance. To explore the Hardy-Weinberg equilibrium (HWE), we consider genotype distribution in control samples and only those variants having HWE p value > 0.05 were utilized in further association analysis.

Further, to explore the functional significant of mutant allele “A” of rs1801706 (c.*84G>A), we utilized the genome expression dataset GSE6536 of HapMap population from GEO (gene expression omnibus) database [17] and genotype data of *CETP* with ±10 kb flanking region from ftp://ftp.ncbi.nlm.nih.gov/hapmap/genotypes/2009-01_phaseIII/plink_format/. Further, we extracted the population-wise normalized expression value of *CETP* specific probe GI_4557442 from above downloaded GSE6536 dataset and performed quantitative trait association analysis using Plink software [16]. To explore the group-wise differences of mRNA level, we performed t-test using R. Moreover, to conclude the relation between higher mRNA concentrations with CAD patients, we utilized the relationship discussed in previous reports. On the basis of Barkowski, RS *et*. *al*. and Tan, MH [18,19];

$${Concentration}_{HDL-C}\propto \frac{1}{{Concentration}_{mRNA-CETP}}$$

$${Risk}_{CAD}\propto \frac{1}{{Concentration}_{HDL-C}}$$ (1)

Hence, the genetic risk of CAD will be directly proportional to the mRNA concentration of *CETP*;

$${Risk}_{CAD}\propto {Concentration}_{mRNA-CETP}$$ (2)

## Results

**Fig 1:**
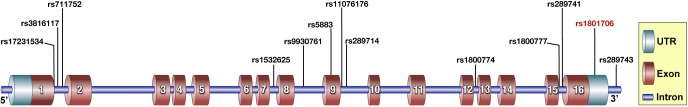
Observed variations and its location in *CETP* gene.mRNA. ENST00000200676 was utilized to represent the physical location of variants.

We have investigated the exons, exon-intron boundaries and UTR of *CETP* in 323 individuals with CAD and 300 ethnically matched controls. In total, we found thirteen variants (SNPs), of which one was in splice regions [rs1532625 (C/T)]; eight were in introns [rs17231534 (C/A/T) and rs3816117 (T/C) rs711752 (G/A),rs9930761 (T/C), rs11076176 (T/G), rs289714 (G/A), rs1800774(C/T) and rs289741(G/A)]; one was synonymous [rs5883(C/T)]; one was missense [rs1800777 (G/A)]; one was in 3’ UTR [rs1801706 (G/A)]; and one was in the downstream position of gene [rs289743 (G/C)] (**Table 3 and Fig 1**).

**Table 3:** Genotype and allele frequency distributions of SNPs in *CETP* among cases and controls.

SNP	Gt/Al^+^	CAD(N/%) = 323	Control(N/%) = 300	OR (95% CI)	P-value	HWE P-value
**rs17231534(C/A)**	CC	263(81.42)	260(86.66)	1.48(0.94–2.35)	0.095	~0
**Intron variant**	CA	12(3.71)	17(5.66)			
	AA	48(14.86)	23(7.66)			
	C	574(88.85)	537(89.5)	1.07(0.74–1.55)	0.783	
	A	72(11.14)	63(10.5)			
**rs3816117(T/C)**	TT	115(35.60)	150(50)	1.81(1.30–2.53)	3.85E-04	~0
**Intron variant**	TC	98(30.34)	24(8)			
	CC	110(34.05)	126(42)			
	T	328(50.77)	324(54)	1.2(0.94–1.52)	0.146	
	C	318(49.22)	276(46)			
**rs711752(G/A)**	GG	169(52.32)	152(50.66)	0.731(0.67–1.30)	0.731	~0
**Intron variant**	GA	89(27.55)	84(28)			
	AA	65(20.12)	64(21.33)			
	G	427(66.09)	388(64.66)	0.94(0.74–1.19)	0.0637	
	A	219(33.90)	212(35.33)			
**rs1532625(C/T)**	CC	99(30.65)	81(27)	0.84(0.58–1.20)	0.36	0.422
**Splice region variant**	CT	128(39.62)	143(47.66)			
	TT	96(29.72)	76(25.33)			
	C	326(50.46)	305(50.83)	0.84(0.58–1.20)	0.36	
	T	320(49.53)	295(49.16)			
**rs9930761 (T/C)**	TT	300(92.87)	282(94)	1.2(0.61–2.38)	0.688	~0
**Intron variant**	TC	22(6.81)	14(4.66)			
	CC	1(0.30)	4(1.33)			
	T	622(96.28)	578(96.33)	1.01(0.54–1.90)	1	
	C	24(3.71)	22(3.66)			
**rs5883(C/T)**	CC	305(94.42)	287(95.66)	1.3(0.59–2.88)	0.599	0.701
**Synonymous variant**	CT	18(5.57)	13(4.33)			
	TT	0(0)	0(0)			
	C	628(97.21)	587(97.83)	1.29(0.60–2.82)	0.603	
	T	18(2.78)	13(2.16)			
**rs11076176 (T/G)**	TT	212(65.63)	192(64)	0.93(0.66–1.31)	0.732	1
**Intron variant**	TG	101(31.26)	96(32)			
	GG	10(3.09)	12(4)			
	T	525(81.26)	480(80)	0.92(0.69–1.23)	0.621	
	G	121(18.73)	120(20)			
**rs289714 (G/A)**	GG	23(7.12)	12(4)	0.54(0.25–1.17)	0.129	0.436
**Intron variant**	GA	98(30.34)	107(35.66)			
	AA	202(62.53)	181(60.33)			
	G	144(22.29)	131(21.83)	0.97(0.74–1.28)	0.99	
	A	502(77.70)	469(78.16)			
**rs1800774(C/T)**	CC	180(55.72)	163(54.33)	0.95(0.68–1.31)	0.788	0.0018
**Intron variant**	CT	108(33.43)	101(33.66)			
	TT	35(10.83)	36(12)			
	C	468(72.44)	427(71.16)	0.87(0.69–1.10)	0.254	
	T	178(27.55)	173(28.83)			
**rs1800777 (G/A)**	GG	309(95.66)	295(98.33)	2.67(0.89–8.61)	0.089	~0
**Missense variant**	GA	14(4.33)	3(1)			
	AA	0(0)	2(0.66)			
	G	632(97.83)	593(98.83)	1.88(0.70–5.17)	0.25	
	A	14(2.16)	7(1.16)			
**rs289741(G/A)**	GG	73(22.60)	58(19.33)	0.82(0.55–1.23)	0.367	0.933
	GA	159(49.22)	147(49)			
	AA	91(28.17)	95(31.66)			
	G	305(47.21)	263(43.83)	0.87(0.69–1.10)	0.254	
	A	341(52.78)	337(56.16)			
rs1801706(G/A) (c.*84G>A)	**GG**	**195(60.37)**	**230(76.66)**	**2.16(1.50–3.10)**	**1.88E-05**	**0.135**
**3 prime UTR variant**						
	**GA**	**112(34.67)**	**62(20.66)**			
	**AA**	**16(4.95)**	**8(2.66)**			
	**G**	**502(77.70)**	**522(87)**	**1.92(1.40–2.63)**	**2.57E-05**	
**rs289743 (G/C)**	**A**	**144(22.29)**	**78(13)**			
**Downstream gene variant**	GG	195(60.37)	183(61)	1.03(0.73–1.44)	0.938	0.000001
	GC	117(36.22)	81(27)			
	CC	11(3.4)	36(12)			
	G	507(78.48)	447(74.5)	0.8(0.61–1.05)	0.111	
	C	139(21.51)	153(25.5)			

^+^Gt/Al- Genotype/Allele

Among these 13 variants, 6 were in HWE equilibrium (p>0.05) (**Table 3**). Of which, rs1801706 (c.*84G>A) was significantly associated with patients group. The 3’ UTR variant c.*84G>A (G/A) showed a genotype distribution (%) of 60.37 (GG), 34.67 (GA) and 4.95 (AA) among individuals with CAD; whereas in controls the frequencies were 76.66 (GG), 20.66 (GA) and 2.66 (AA). Association analysis with genotype showed significant association with CAD (OR = 2.16, 95% CI: 1.50–3.10, p = 1.88x10^-5^). The allelic distribution showed 77.70% of ‘G’ and 22.29% of ‘A’ in cases and among the controls the G showed 87% and ‘A’ showed 13% with significant association of the ‘A’ allele with OR = 1.92, 95% CI; 1.40–2.63, p-value 2.57x10^-5^. Both genotype and allele distribution of *CETP* SNPs among cases and controls are given in **Table 3**.Since, we did not observed LD differences between the cases and controls; we did not proceed for haplotype analysis.

### 3.1. Functional validation of rs1801706/c.*84G>A

**Fig 2:**
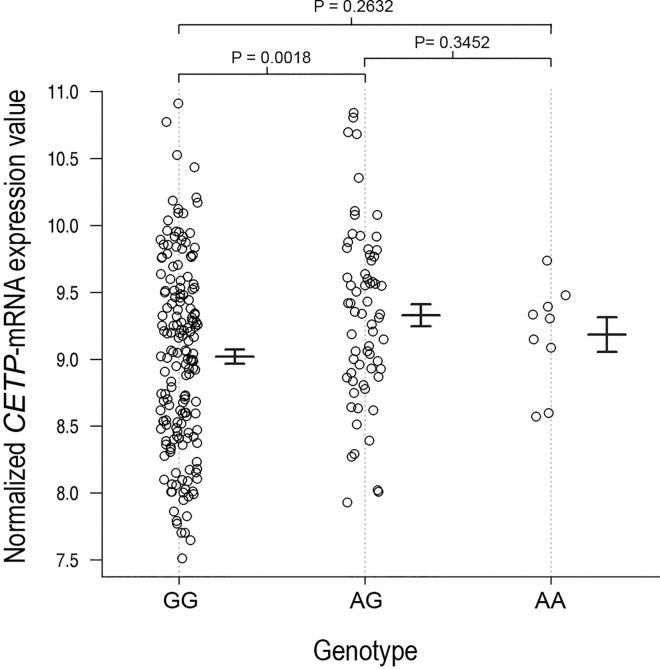
Expression data of *CETP* in HapMap population according to their genotype. Middle bar represents the mean value of expression while flanking bar represents their standard deviation.

To functionally validate the mutant allele “A” (c.*84G>A), we have utilized the whole genome gene-expression data of 210 HapMap samples [17, 20] and performed QTL analysis with genotype information of same individuals from HapMap project, with additive model (**Table 4**) and observed variant rs1801706 in association with *CETP* mRNA level with p-value 7.09×10^−3^ (R^2^ = 0.029 and β = 0.2163) (**Table 4**). The genotype and normalized mRNA intensity/expression value for c.*84G>A is given in **S1 Table**. Interestingly, both heterozygous (GA) and mutant homozygous (AA) genotype were found to influence the higher level of mRNA (**Fig 2**).We also explored pair-wise comparison of mRNA expression between genotypes and observed that mRNA expression level of genotype AA *vs*. AG (p-value = 0.3452) and genotype AA *vs*. GG (p-value = 0.2632) were not significant, while genotype AG *vs*. GG was significantly different (p-value = 0.0018).

**Table 4:** SNPs present within ±10kb of *CETP* and their respective association p-value with normalized mRNA expression level. Only rs1801706 (c.*84G>A) was significantly associated and highlighted in bold.

Chr.	SNP	Physical position (hg19)	No. of subjects	β	S.E.	R2	T	P value
16	rs247617	56990716	247	-0.0726	0.0691	0.0045	-1.0510	0.2943
16	rs6499863	56992017	247	0.0721	0.0739	0.0039	0.9758	0.3301
16	rs3764261	56993324	247	-0.1114	0.0658	0.0116	-1.6940	0.0916
16	rs12447924	56994192	245	-0.0866	0.0754	0.0054	-1.1490	0.2517
16	rs4783961	56994894	247	-0.0268	0.0605	0.0008	-0.4437	0.6577
16	rs4783962	56995038	247	0.0309	0.0788	0.0006	0.3914	0.6959
16	rs1800775	56995236	247	0.0567	0.0621	0.0034	0.9127	0.3623
16	rs1864163	56997233	242	-0.0122	0.0750	0.0001	-0.1622	0.8713
16	rs7203984	56999258	246	-0.1174	0.0590	0.0160	-1.9890	0.0478
16	rs12597002	57002404	247	0.0600	0.0692	0.0031	0.8676	0.3864
16	rs891142	57003977	246	-0.0751	0.1151	0.0017	-0.6527	0.5146
16	rs12720860	57004662	247	-0.3030	0.2037	0.0090	-1.4880	0.1382
16	rs7205804	57004889	241	-0.0097	0.0631	0.0001	-0.1542	0.8776
16	rs1532624	57005479	247	-0.0014	0.0633	0.0000	-0.0217	0.9827
16	rs12708974	57005550	247	0.1446	0.1023	0.0081	1.4130	0.1588
16	rs12720872	57005882	247	-0.2828	0.1833	0.0096	-1.5420	0.1243
16	rs7499892	57006590	247	-0.0666	0.0713	0.0035	-0.9338	0.3513
16	rs9930761	57007192	247	0.2321	0.1142	0.0166	2.0320	0.0432
16	rs5883	57007353	247	0.2106	0.1218	0.0121	1.7290	0.0850
16	rs289714	57007451	246	-0.0752	0.0615	0.0061	-1.2220	0.2230
16	rs12691052	57007512	247	0.0782	0.1787	0.0008	0.4374	0.6622
16	rs289715	57008508	247	-0.0864	0.0757	0.0053	-1.1410	0.2549
16	rs4784744	57011185	247	0.0464	0.0627	0.0022	0.7403	0.4598
16	rs12720898	57011243	247	0.1187	0.1088	0.0048	1.0910	0.2764
16	rs891144	57011936	247	0.0548	0.1076	0.0011	0.5094	0.6109
16	rs12708980	57012379	247	-0.1229	0.0669	0.0136	-1.8370	0.0675
16	rs7195984	57015463	247	-0.0709	0.0989	0.0021	-0.7172	0.4739
16	rs5882	57016092	247	-0.0677	0.0586	0.0054	-1.1560	0.2487
16	rs5742907	57016150	247	-0.7002	0.6914	0.0042	-1.0130	0.3122
16	rs12596364	57016519	247	0.0216	0.1514	0.0001	0.1425	0.8868
16	rs289740	57016950	244	-0.0694	0.1125	0.0016	-0.6172	0.5377
16	rs9923854	57017002	247	0.2583	0.1044	0.0244	2.4750	0.0140
16	rs2228667	57017279	247	0.0936	0.6928	0.0001	0.1350	0.8927
16	rs2303790	57017292	245	-0.2835	0.2478	0.0054	-1.1440	0.2537
16	rs1800777	57017319	246	-0.0417	0.2336	0.0001	-0.1783	0.8586
16	rs5887	57017552	243	-0.2041	0.2455	0.0029	-0.8316	0.4065
**16**	**rs1801706**	**57017662**	**247**	**0.2163**	**0.0797**	**0.0292**	**2.7150**	**0.0071**
16	rs289742	57017762	247	-0.0577	0.0659	0.0031	-0.8752	0.3823
16	rs289744	57018102	247	0.0483	0.0588	0.0027	0.8209	0.4125
16	rs12720917	57019392	247	-0.0059	0.1335	0.0000	-0.0442	0.9648
16	rs289745	57019532	247	-0.0332	0.0614	0.0012	-0.5402	0.5895
16	rs12934552	57021433	246	-0.0941	0.1237	0.0024	-0.7610	0.4474
16	rs289747	57023938	247	0.0033	0.0659	0.0000	0.0498	0.9603
16	rs17290922	57024317	247	-0.0919	0.1334	0.0019	-0.6887	0.4917
16	rs1566439	57024662	247	0.0181	0.0590	0.0004	0.3075	0.7587

### 3.2. Prevalence of rs1801706/c.*84G>A

Further, we explored the frequency spectrum of rs1801706 (c.*84G>A) in other world populations, including Indians. We utilized the genotype dataset from 1000 genome project. We observed that only 3 populations were having frequency of <0.1 forrs1801706-A variant; (1) 0.086 in CDX (Chinese Dai), (2) 0.078 in MXL (Mexicans) and (3) 0.029 in PEL (Peruvians) (**Table 5).**

**Table 5:** Frequency spectrum of allele and genotype of rs1801706/ c. *84G>A in case, control and different world populations.

Population	Allele frequency (count)		Genotype frequency (count)		
	Wild type allele"G"	Mutant allele"A"	GG	AG	AA
**Case**	0.777 (502)	0.2229 (144)	0.6037 (195)	0.3467 (112)	0.0495 (16)
**Control**	0.87 (522)	0.13 (78)	0.7666 (230)	0.2066 (62)	0.0266 (8)
**All population**	0.845 (4230)	0.155 (778)	0.713 (1785)	0.264 (660)	0.024 (59)
**South-Asians**	0.786 (769)	0.214 (209)	0.607 (297)	0.358 (175)	0.035 (17)
BEB (Bengali)	0.791 (136)	0.209 (36)	0.605 (52)	0.372 (32)	0.023 (2)
GIH (Guajarati Indians)	0.801 (165)	0.199 (41)	0.612 (63)	0.379 (39)	0.010 (1)
ITU (Telugu)	0.775 (158)	0.225 (46)	0.588 (60)	0.373 (38)	0.039 (4)
PJL (Punjabi)	0.771 (148)	0.229 (44)	0.583 (56)	0.375 (36)	0.042 (4)
STU (SriLankan Tamil)	0.794 (162)	0.206 (42)	0.647 (66)	0.294 (30)	0.059 (6)
**East-Asians**	0.895 (902)	0.105 (106)	0.810 (408)	0.171 (86)	0.020 (10)
CDX (Chinese Dai)	0.914 (170)	0.086 (16)	0.828 (77)	0.172 (16)	0.0 (0))
CHB (Han Chinese)	0.883 (182)	0.117 (24)	0.796 (82)	0.175 (18)	0.029 (3)
CHS (Southern Han Chinese)	0.900 (189)	0.100 (21)	0.829 (87)	0.143 (15)	0.029 (3)
JPT (Japanese)	0.880 (183)	0.120 (25)	0.788 (82)	0.183 (19)	0.029 (3)
KHV (Kinh)	0.899 (178)	0.101 (20)	0.808 (80)	0.182 (18)	0.010 (1)
**African**	0.846 (1119)	0.154 (203)	0.717 (474)	0.259 (171)	0.024 (16)
ACB (Caribbeans)	0.854 (164)	0.146 (28)	0.719 (69)	0.271 (26)	0.010 (1)
ASW (Afro-Americans)	0.885 (108)	0.115 (14)	0.770 (47)	0.230 (14)	0.0 (0))
ESN (Esan)	0.843 (167)	0.157 (31)	0.707 (70)	0.273 (27)	0.020 (2)
LWK (Luhya)	0.808 (160)	0.192 (38)	0.657 (65)	0.303 (30)	0.040 (4)
MAG (Mandinka)	0.858 (194)	0.142 (32)	0.761 (86)	0.195 (22)	0.044 (5)
MSL(Mende)	0.865 (147)	0.135 (23)	0.741 (63)	0.247 (21)	0.012 (1)
YRI (Yoruba)	0.829 (179)	0.171 (37)	0.685 (74)	0.287 (31)	0.028 (3)
**American**	0.905 (628)	0.095 (66)	0.821 (285)	0.167 (58)	0.012 (4)
CLM (Colombians)	0.878 (165)	0.122 (23)	0.787 (74)	0.181 (17)	0.032 (3)
MXL(Mexicans)	0.922 (118)	0.078 (10)	0.844 (54)	0.156 (10)	0.0 (0))
PEL (Peruvians)	0.971 (165)	0.029 (5)	0.941 (80)	0.059 (5)	0.0 (0))
PUR (Puerto Ricans)	0.865 (180)	0.135 (28)	0.740 (77)	0.250 (26)	0.010 (1)
**Europeans**	0.807 (812)	0.193 (194)	0.638 (321)	0.338 (170)	0.024 (12)
CEU (Utah residents)	0.778 (154)	0.222 (44)	0.586 (58)	0.384 (38)	0.030 (3)
FIN (Finnish)	0.813 (161)	0.187 (37)	0.657 (65)	0.313 (31)	0.030 (3)
GBR (British)	0.797 (145)	0.203 (37)	0.626 (57)	0.341 (31)	0.033 (3)
IBS (Iberian)	0.822 (176)	0.178 (38)	0.654 (70)	0.336 (36)	0.009 (1)
TSI (Toscani)	0.822 (176)	0.178 (38)	0.664 (71)	0.318 (34)	0.019 (2)

## Discussion

CAD is caused by multiple genetic and environmental factors [2].*CETP* plays a central role in human lipoprotein metabolism, as it facilitates the removal of excess cholesterol from the body via LDL receptor-mediated uptake in the liver and excretion into the bile [21]. Our earlier study on the -629 promoter of *CETP* gene had shown a significant association between CAD patients and controls [22]. Considering the crucial role of *CETP* in lipid metabolism, we investigated the association of genetic variants of the *CETP* with risk of coronary artery disease in patients from South India.

In the present study, we observed a 3’ UTR variant, rs1801706 (c.*84G>A) is associated with the CAD in South Indians, however, we did not find association of a few previously reported SNPs. A genome-wide linkage analysis conducted on healthy American woman cohort had analyzed over 350,000 SNPs and found only SNPs flanking or in the *CETP* gene were associated with both HDL-C and risk of incident CAD [23].Papp *et*.*al*. observed that rs5883*T*/rs9930761*C* was associated with increased HDL-C levels in males [24]. Although our study group had 76% males, we did not find any significant association with rs5883. Studies on C>T/In9 (rs289714) was earlier shown to be associated with undesirable changes in adiposity and HDL-C levels when exposed to excessive calorie consumption [25,26], however, we did not find significant association between cases and the controls. Studies on Caucasians and African Americans [27] showed association of *CETP* variations with myocardial infarction (MI).

The rs1800777 (Arg 451Gln) is located within the lipid-binding region of *CETP* protein and possibly may result in the loss of positive charge, varying the binding efficiency of *CETP* to cholesteryl esters. Lu *et al*. reported that rs1800777was associated with lower plasma HDL cholesterol levels [28], whereas Moleres *et al*. reported that this SNP is strongly associated with adiposity indexes [29]. However, in the present study both the genotype and allele frequency of this variant did not show any association with CAD. Interestingly, we found rs1801706 (c.*84G>A) was significantly associated with CAD, which is in agreement with Whitehall II *et al*.

Since, associated variant rs1801706 (c.*84G>A) was observed in UTR region, we predicted that mutant allele “A” might be affecting mRNA expression of *CETP*. Here, we were not aware that mutant allele “A” increasing or decreasing the expression. But, using Eq 2, it can be further predicted that mutant allele “A” should increase the expression because risk of CAD increases with expression level and patients were having higher frequency of “A”. Interestingly, in QTL analysis, we observed that our prediction is true.

It is well known that inhibition of *CETP* decrease LDL level while increase the HDL, vice-versa might be true. We can predict that high expression of *CETP*, due to mutant allele “A”, is responsible for high LDL level in CAD patients. This might be the reason of plague formation in blood vessels and further causing coronary artery disease.

Further, data obtained for rs1801706 (c.*84G>A) from the 1000 genome project revealed that only 3 populations were having frequency of<0.1; (1) 0.086 in CDX (Chinese Dai), (2) 0.078 in MXL (Mexicans) and (3) 0.029 in PEL (Peruvians) (**Table 5**). Populations with Indian ancestry (GIH, STU, ITU and BEB) have higher frequency of this allele compared to the controls. This might be due to the admixture of migrant Indians with local populations, who might have higher frequency of rs1801706-A allele (Europeans population in 1000 genome project).

## Conclusion

In conclusion, our study revealed rs1801706 (c.*84G>A), a functionally relevant variant in 3’ UTR of *CETP*, is strongly associated with CAD in South Indian. Interestingly, mutant allele “A” was found to be associated with higher concentration of *CETP* mRNA. Since, *CETP* involve in the conversion of HDL to LDL/VLDL, we are tempted to conclude that rs1801706-A increases the risk of CAD by increasing rate of conversion of HDL to LDL/VLDL, through changing the half life of *CETP* mRNA.

## Supporting Information

S1 Table Details of normalized expression value of *CETP* mRNA with genotype of rs1801706/c. *84G>A in same samples of HapMap populations.
(DOCX)
