# Supplementary material for: c.*84G>A Mutation in CETP Is Associated with Coronary Artery Disease in South Indians
Source: PLoS One. 2016 Oct 21;11(10):e0164151. doi: 10.1371/journal.pone.0164151 (PMC5074517; doi:10.1371/journal.pone.0164151)
Supplement: S1 Table — *84G>A in same samples of HapMap populations. (DOCX) [file pone.0164151.s001.docx]

**S1table**: Details of normalized expression value of *CETP* mRNA with genotype of rs1801706/c.*84G>A in same samples of HapMap populations

| Continent | Number | HapMap populations | Family ID | Individual ID | Normalized intensity value | Genotype |
| --- | --- | --- | --- | --- | --- | --- |
| European | 1 | CEU | 1444 | NA12750 | 9.6101 | *AG* |
| European | 2 | CEU | 1350 | NA11831 | 9.57957 | *AG* |
| European | 3 | CEU | 1334 | NA12146 | 8.86791 | *AG* |
| European | 4 | CEU | 1347 | NA11882 | 8.68252 | *GG* |
| European | 5 | CEU | 1340 | NA07056 | 9.04913 | *GG* |
| European | 6 | CEU | 1358 | NA12707 | 9.81803 | *AG* |
| European | 7 | CEU | 1408 | NA12154 | 9.58786 | *GG* |
| European | 8 | CEU | 1447 | NA12753 | 8.15166 | *GG* |
| European | 9 | CEU | 1349 | NA11839 | 10.0932 | *GG* |
| European | 10 | CEU | 1347 | NA10859 | 9.42054 | *AG* |
| European | 11 | CEU | 1459 | NA12875 | 9.3407 | *AG* |
| European | 12 | CEU | 1345 | NA07348 | 8.5752 | *GG* |
| European | 13 | CEU | 1408 | NA12156 | 9.20647 | *GG* |
| European | 14 | CEU | 1346 | NA12044 | 8.99597 | *GG* |
| European | 15 | CEU | 1362 | NA11992 | 10.2091 | *GG* |
| European | 16 | CEU | 1350 | NA11829 | 8.57087 | *AA* |
| European | 17 | CEU | 1334 | NA12239 | 8.83325 | *GG* |
| European | 18 | CEU | 1447 | NA12762 | 8.38885 | *GG* |
| European | 19 | CEU | 1358 | NA12716 | 10.0948 | *GG* |
| European | 20 | CEU | 1463 | NA12878 | 8.53737 | *GG* |
| European | 21 | CEU | 1350 | NA10856 | 9.47934 | *AA* |
| European | 22 | CEU | 1459 | NA12874 | 10.9135 | *GG* |
| European | 23 | CEU | 1447 | NA12760 | 9.73917 | *AG* |
| European | 24 | CEU | 1341 | NA06985 | 9.59785 | *AG* |
| European | 25 | CEU | 1420 | NA12003 | 8.46012 | *GG* |
| European | 26 | CEU | 1416 | NA10835 | 8.40076 | *GG* |
| European | 27 | CEU | 1340 | NA07022 | 9.25897 | *GG* |
| European | 28 | CEU | 1454 | NA12813 | 9.4198 | *AG* |
| European | 29 | CEU | 1420 | NA10839 | 8.93003 | *AG* |
| European | 30 | CEU | 1341 | NA07055 | 9.06555 | *GG* |
| European | 31 | CEU | 1344 | NA12056 | 8.92652 | *GG* |
| European | 32 | CEU | 1375 | NA10863 | 9.55167 | *AG* |
| European | 33 | CEU | 1334 | NA12145 | 9.16679 | *GG* |
| European | 34 | CEU | 1454 | NA12814 | 9.43066 | *AG* |
| European | 35 | CEU | 1334 | NA10847 | 8.08998 | *GG* |
| European | 36 | CEU | 1420 | NA12006 | 9.2516 | *GG* |
| European | 37 | CEU | 1447 | NA12763 | 9.94433 | *GG* |
| European | 38 | CEU | 1345 | NA07357 | 8.54054 | *GG* |
| European | 39 | CEU | 1334 | NA12144 | 9.96276 | *GG* |
| European | 40 | CEU | 1408 | NA10831 | 8.47972 | *GG* |
| European | 41 | CEU | 1340 | NA07000 | 8.42525 | *GG* |
| European | 42 | CEU | 1350 | NA11832 | 9.50511 | *AG* |
| European | 43 | CEU | 1341 | NA06991 | 8.60121 | *GG* |
| European | 44 | CEU | 1349 | NA11840 | 10.0789 | *AG* |
| European | 45 | CEU | 1454 | NA12802 | 10.6819 | *AG* |
| European | 46 | CEU | 1447 | NA12761 | 9.87362 | *GG* |
| European | 47 | CEU | 1408 | NA10830 | 9.30982 | *GG* |
| European | 48 | CEU | 1350 | NA10855 | 8.00962 | *AG* |
| European | 49 | CEU | 1340 | NA06994 | 9.76861 | *AG* |
| European | 50 | CEU | 1362 | NA11993 | 9.52257 | *GG* |
| European | 51 | CEU | 1362 | NA11995 | 9.31044 | *AG* |
| European | 52 | CEU | 1463 | NA12891 | 8.02647 | *GG* |
| European | 53 | CEU | 1459 | NA12864 | 9.56297 | *AG* |
| European | 54 | CEU | 1444 | NA12751 | 8.59643 | *GG* |
| European | 55 | CEU | 1362 | NA10861 | 8.80617 | *AG* |
| European | 56 | CEU | 1420 | NA12005 | 9.83167 | *AG* |
| European | 57 | CEU | 1375 | NA12234 | 9.73808 | *AA* |
| European | 58 | CEU | 1345 | NA07345 | 9.28987 | *GG* |
| European | 59 | CEU | 1340 | NA07029 | 8.39177 | *AG* |
| European | 60 | CEU | 1463 | NA12892 | 8.42034 | *GG* |
| European | 61 | CEU | 1416 | NA12248 | 8.74038 | *GG* |
| European | 62 | CEU | 1334 | NA10846 | 7.51062 | *GG* |
| European | 63 | CEU | 1454 | NA12801 | 7.93098 | *AG* |
| European | 64 | CEU | 1459 | NA12872 | 7.99122 | *GG* |
| European | 65 | CEU | 1408 | NA12155 | 9.37939 | *GG* |
| European | 66 | CEU | 1341 | NA06993 | 7.86098 | *GG* |
| European | 67 | CEU | 1350 | NA11830 | 9.20828 | *AG* |
| European | 68 | CEU | 1420 | NA10838 | 8.01139 | *GG* |
| European | 69 | CEU | 1416 | NA12249 | 9.87677 | *AG* |
| European | 70 | CEU | 1344 | NA12057 | 8.83648 | *AG* |
| European | 71 | CEU | 1454 | NA12812 | 7.77198 | *GG* |
| European | 72 | CEU | 1347 | NA11881 | 10.8422 | *AG* |
| European | 73 | CEU | 1362 | NA11994 | 9.56115 | *GG* |
| European | 74 | CEU | 1459 | NA12873 | 9.33382 | *AA* |
| European | 75 | CEU | 1454 | NA12815 | 9.6929 | *GG* |
| European | 76 | CEU | 1444 | NA12740 | 10.17 | *GG* |
| European | 77 | CEU | 1447 | NA12752 | 7.64599 | *GG* |
| European | 78 | CEU | 1346 | NA12043 | 8.62004 | *GG* |
| European | 79 | CEU | 1375 | NA12264 | 8.79245 | *GG* |
| European | 80 | CEU | 1349 | NA10854 | 8.87982 | *GG* |
| European | 81 | CEU | 1459 | NA12865 | 8.95901 | *AG* |
| East-Asians | 1 | CHB | NA18524 | NA18524 | 10.5272 | *GG* |
| East-Asians | 2 | CHB | NA18635 | NA18635 | 9.2215 | *GG* |
| East-Asians | 3 | CHB | NA18537 | NA18537 | 9.48743 | *GG* |
| East-Asians | 4 | CHB | NA18572 | NA18572 | 9.05997 | *AG* |
| East-Asians | 5 | CHB | NA18592 | NA18592 | 9.05635 | *GG* |
| East-Asians | 6 | CHB | NA18526 | NA18526 | 9.25226 | *GG* |
| East-Asians | 7 | CHB | NA18529 | NA18529 | 10.1858 | *GG* |
| East-Asians | 8 | CHB | NA18558 | NA18558 | 9.84162 | *GG* |
| East-Asians | 9 | CHB | NA18562 | NA18562 | 9.2441 | *GG* |
| East-Asians | 10 | CHB | NA18545 | NA18545 | 10.3567 | *AG* |
| East-Asians | 11 | CHB | NA18609 | NA18609 | 9.19997 | *GG* |
| East-Asians | 12 | CHB | NA18552 | NA18552 | 9.51355 | *GG* |
| East-Asians | 13 | CHB | NA18611 | NA18611 | 8.93515 | *GG* |
| East-Asians | 14 | CHB | NA18555 | NA18555 | 9.21068 | *GG* |
| East-Asians | 15 | CHB | NA18566 | NA18566 | 9.21581 | *GG* |
| East-Asians | 16 | CHB | NA18563 | NA18563 | 9.48263 | *GG* |
| East-Asians | 17 | CHB | NA18570 | NA18570 | 9.46109 | *GG* |
| East-Asians | 18 | CHB | NA18612 | NA18612 | 9.7061 | *GG* |
| East-Asians | 19 | CHB | NA18621 | NA18621 | 9.83393 | *GG* |
| East-Asians | 20 | CHB | NA18622 | NA18622 | 9.39405 | *AA* |
| East-Asians | 21 | CHB | NA18573 | NA18573 | 10.1248 | *GG* |
| East-Asians | 22 | CHB | NA18577 | NA18577 | 7.94585 | *GG* |
| East-Asians | 23 | CHB | NA18579 | NA18579 | 9.46255 | *GG* |
| East-Asians | 24 | CHB | NA18632 | NA18632 | 9.91759 | *GG* |
| East-Asians | 25 | CHB | NA18636 | NA18636 | 8.61867 | *GG* |
| East-Asians | 26 | CHB | NA18593 | NA18593 | 9.60071 | *GG* |
| East-Asians | 27 | CHB | NA18603 | NA18603 | 9.63789 | *GG* |
| East-Asians | 28 | CHB | NA18624 | NA18624 | 9.95095 | *GG* |
| East-Asians | 29 | CHB | NA18550 | NA18550 | 9.76303 | *GG* |
| East-Asians | 30 | CHB | NA18605 | NA18605 | 8.34062 | *GG* |
| East-Asians | 31 | CHB | NA18542 | NA18542 | 8.1012 | *GG* |
| East-Asians | 32 | CHB | NA18532 | NA18532 | 9.35422 | *AG* |
| East-Asians | 33 | CHB | NA18561 | NA18561 | 9.38297 | *GG* |
| East-Asians | 34 | CHB | NA18608 | NA18608 | 9.14809 | *AG* |
| East-Asians | 35 | CHB | NA18564 | NA18564 | 9.137 | *GG* |
| East-Asians | 36 | CHB | NA18571 | NA18571 | 9.33289 | *GG* |
| East-Asians | 37 | CHB | NA18620 | NA18620 | 8.61826 | *AG* |
| East-Asians | 38 | CHB | NA18623 | NA18623 | 8.9881 | *GG* |
| East-Asians | 39 | CHB | NA18576 | NA18576 | 8.6767 | *GG* |
| East-Asians | 40 | CHB | NA18582 | NA18582 | 8.98792 | *AG* |
| East-Asians | 41 | CHB | NA18633 | NA18633 | 8.89033 | *GG* |
| East-Asians | 42 | CHB | NA18637 | NA18637 | 9.91814 | *AG* |
| East-Asians | 43 | CHB | NA18594 | NA18594 | 8.10663 | *GG* |
| East-Asians | 44 | JPT | NA18942 | NA18942 | 8.37407 | *GG* |
| East-Asians | 45 | JPT | NA18949 | NA18949 | 9.34253 | *GG* |
| East-Asians | 46 | JPT | NA18970 | NA18970 | 9.09962 | *AG* |
| East-Asians | 47 | JPT | NA18945 | NA18945 | 8.48712 | *GG* |
| East-Asians | 48 | JPT | NA18940 | NA18940 | 9.16026 | *GG* |
| East-Asians | 49 | JPT | NA18964 | NA18964 | 9.18729 | *AG* |
| East-Asians | 50 | JPT | NA18953 | NA18953 | 9.27666 | *GG* |
| East-Asians | 51 | JPT | NA18961 | NA18961 | 9.76537 | *GG* |
| East-Asians | 52 | JPT | NA18972 | NA18972 | 8.41054 | *GG* |
| East-Asians | 53 | JPT | NA18967 | NA18967 | 8.27755 | *GG* |
| East-Asians | 54 | JPT | NA18976 | NA18976 | 8.06379 | *GG* |
| East-Asians | 55 | JPT | NA18981 | NA18981 | 8.59578 | *GG* |
| East-Asians | 56 | JPT | NA18971 | NA18971 | 8.02013 | *AG* |
| East-Asians | 57 | JPT | NA18994 | NA18994 | 8.52928 | *GG* |
| East-Asians | 58 | JPT | NA18998 | NA18998 | 9.7782 | *AG* |
| East-Asians | 59 | JPT | NA19000 | NA19000 | 9.49879 | *GG* |
| East-Asians | 60 | JPT | NA18943 | NA18943 | 9.04085 | *AG* |
| East-Asians | 61 | JPT | NA18947 | NA18947 | 9.43153 | *GG* |
| East-Asians | 62 | JPT | NA18944 | NA18944 | 9.0092 | *GG* |
| East-Asians | 63 | JPT | NA18948 | NA18948 | 8.40948 | *GG* |
| East-Asians | 64 | JPT | NA18951 | NA18951 | 9.33588 | *AG* |
| East-Asians | 65 | JPT | NA18952 | NA18952 | 9.04352 | *GG* |
| East-Asians | 66 | JPT | NA18956 | NA18956 | 9.30565 | *AA* |
| East-Asians | 67 | JPT | NA18968 | NA18968 | 9.61662 | *GG* |
| East-Asians | 68 | JPT | NA18959 | NA18959 | 9.08544 | *AA* |
| East-Asians | 69 | JPT | NA18969 | NA18969 | 8.9485 | *GG* |
| East-Asians | 70 | JPT | NA18960 | NA18960 | 9.85558 | *GG* |
| East-Asians | 71 | JPT | NA18965 | NA18965 | 8.59817 | *AA* |
| East-Asians | 72 | JPT | NA18973 | NA18973 | 9.25662 | *GG* |
| East-Asians | 73 | JPT | NA18966 | NA18966 | 8.72048 | *GG* |
| East-Asians | 74 | JPT | NA18975 | NA18975 | 9.00284 | *GG* |
| East-Asians | 75 | JPT | NA18978 | NA18978 | 9.37038 | *GG* |
| East-Asians | 76 | JPT | NA18980 | NA18980 | 8.18048 | *GG* |
| East-Asians | 77 | JPT | NA18974 | NA18974 | 9.28167 | *GG* |
| East-Asians | 78 | JPT | NA18987 | NA18987 | 8.99567 | *GG* |
| East-Asians | 79 | JPT | NA18990 | NA18990 | 9.53343 | *GG* |
| East-Asians | 80 | JPT | NA18991 | NA18991 | 9.06655 | *GG* |
| East-Asians | 81 | JPT | NA18995 | NA18995 | 8.36231 | *GG* |
| East-Asians | 82 | JPT | NA18997 | NA18997 | 8.72968 | *GG* |
| East-Asians | 83 | JPT | NA19005 | NA19005 | 9.92362 | *AG* |
| East-Asians | 84 | JPT | NA18999 | NA18999 | 8.51897 | *GG* |
| East-Asians | 85 | JPT | NA19007 | NA19007 | 10.4364 | *GG* |
| Africans | 1 | YRI | Y005 | NA18505 | 7.79193 | *GG* |
| Africans | 2 | YRI | Y045 | NA19202 | 9.8573 | *GG* |
| Africans | 3 | YRI | Y117 | NA19240 | 7.70162 | *GG* |
| Africans | 4 | YRI | Y117 | NA19239 | 9.0553 | *GG* |
| Africans | 5 | YRI | Y004 | NA18501 | 9.41541 | *GG* |
| Africans | 6 | YRI | Y043 | NA19137 | 8.99239 | *GG* |
| Africans | 7 | YRI | Y072 | NA19153 | 9.91757 | *GG* |
| Africans | 8 | YRI | Y023 | NA18857 | 7.82692 | *GG* |
| Africans | 9 | YRI | Y058 | NA19223 | 8.74257 | *GG* |
| Africans | 10 | YRI | Y004 | NA18500 | 7.70407 | *GG* |
| Africans | 11 | YRI | Y024 | NA18861 | 9.26069 | *GG* |
| Africans | 12 | YRI | Y045 | NA19201 | 8.15291 | *GG* |
| Africans | 13 | YRI | Y074 | NA19144 | 8.6579 | *GG* |
| Africans | 14 | YRI | Y112 | NA19193 | 9.53533 | *GG* |
| Africans | 15 | YRI | Y005 | NA18503 | 9.76905 | *GG* |
| Africans | 16 | YRI | Y005 | NA18504 | 8.09649 | *GG* |
| Africans | 17 | YRI | Y048 | NA19203 | 8.70162 | *GG* |
| Africans | 18 | YRI | Y074 | NA19143 | 8.54595 | *GG* |
| Africans | 19 | YRI | Y017 | NA18871 | 9.95644 | *GG* |
| Africans | 20 | YRI | Y017 | NA18870 | 9.31887 | *GG* |
| Africans | 21 | YRI | Y045 | NA19200 | 9.19617 | *GG* |
| Africans | 22 | YRI | Y013 | NA18517 | 9.56611 | *AG* |
| Africans | 23 | YRI | Y058 | NA19221 | 9.77926 | *GG* |
| Africans | 24 | YRI | Y024 | NA18863 | 9.51173 | *GG* |
| Africans | 25 | YRI | Y023 | NA18855 | 8.05985 | *GG* |
| Africans | 26 | YRI | Y050 | NA19209 | 8.92281 | *GG* |
| Africans | 27 | YRI | Y056 | NA19160 | 8.30732 | *GG* |
| Africans | 28 | YRI | Y072 | NA19152 | 8.68835 | *GG* |
| Africans | 29 | YRI | Y042 | NA19102 | 8.74734 | *AG* |
| Africans | 30 | YRI | Y047 | NA19172 | 8.00887 | *GG* |
| Africans | 31 | YRI | Y042 | NA19103 | 9.17656 | *GG* |
| Africans | 32 | YRI | Y105 | NA19099 | 9.93679 | *AG* |
| Africans | 33 | YRI | Y018 | NA18854 | 9.89037 | *GG* |
| Africans | 34 | YRI | Y013 | NA18515 | 9.26204 | *AG* |
| Africans | 35 | YRI | Y117 | NA19238 | 9.06099 | *AG* |
| Africans | 36 | YRI | Y071 | NA19142 | 9.63702 | *AG* |
| Africans | 37 | YRI | Y028 | NA18913 | 7.97415 | *GG* |
| Africans | 38 | YRI | Y043 | NA19139 | 9.82714 | *GG* |
| Africans | 39 | YRI | Y028 | NA18912 | 8.00237 | *GG* |
| Africans | 40 | YRI | Y058 | NA19222 | 8.46999 | *GG* |
| Africans | 41 | YRI | Y009 | NA18508 | 9.3247 | *GG* |
| Africans | 42 | YRI | Y077 | NA19129 | 9.02277 | *GG* |
| Africans | 43 | YRI | Y112 | NA19194 | 10.6966 | *AG* |
| Africans | 44 | YRI | Y072 | NA19154 | 10.0374 | *GG* |
| Africans | 45 | YRI | Y043 | NA19138 | 9.44425 | *GG* |
| Africans | 46 | YRI | Y018 | NA18852 | 8.50899 | *GG* |
| Africans | 47 | YRI | Y060 | NA19120 | 9.82179 | *AG* |
| Africans | 48 | YRI | Y050 | NA19210 | 10.1081 | *AG* |
| Africans | 49 | YRI | Y048 | NA19204 | 8.23236 | *GG* |
| Africans | 50 | YRI | Y009 | NA18507 | 8.32756 | *GG* |
| Africans | 51 | YRI | Y056 | NA19159 | 8.77746 | *AG* |
| Africans | 52 | YRI | Y050 | NA19211 | 9.8928 | *GG* |
| Africans | 53 | YRI | Y013 | NA18516 | 8.00881 | *GG* |
| Africans | 54 | YRI | Y024 | NA18862 | 8.96368 | *GG* |
| Africans | 55 | YRI | Y042 | NA19101 | 8.59994 | *GG* |
| Africans | 56 | YRI | Y017 | NA18872 | 8.17395 | *GG* |
| Africans | 57 | YRI | Y112 | NA19192 | 9.55195 | *AG* |
| Africans | 58 | YRI | Y012 | NA18858 | 8.44969 | *GG* |
| Africans | 59 | YRI | Y071 | NA19141 | 8.89779 | *AG* |
| Africans | 60 | YRI | Y040 | NA19093 | 9.19261 | *GG* |
| Africans | 61 | YRI | Y101 | NA19132 | 8.27 | *AG* |
| Africans | 62 | YRI | Y051 | NA19206 | 8.51197 | *AG* |
| Africans | 63 | YRI | Y009 | NA18506 | 9.78782 | *GG* |
| Africans | 64 | YRI | Y105 | NA19098 | 10.0901 | *GG* |
| Africans | 65 | YRI | Y101 | NA19130 | 8.90784 | *GG* |
| Africans | 66 | YRI | Y077 | NA19128 | 8.36088 | *GG* |
| Africans | 67 | YRI | Y101 | NA19131 | 9.14866 | *AA* |
| Africans | 68 | YRI | Y051 | NA19208 | 10.0795 | *AG* |
| Africans | 69 | YRI | Y060 | NA19116 | 8.70246 | *GG* |
| Africans | 70 | YRI | Y028 | NA18914 | 10.7743 | *GG* |
| Africans | 71 | YRI | Y077 | NA19127 | 8.86347 | *AG* |
| Africans | 72 | YRI | Y040 | NA19094 | 10.8076 | *AG* |
| Africans | 73 | YRI | Y056 | NA19161 | 8.63198 | *AG* |
| Africans | 74 | YRI | Y018 | NA18853 | 9.23524 | *GG* |
| Africans | 75 | YRI | Y047 | NA19173 | 9.75962 | *GG* |
| Africans | 76 | YRI | Y047 | NA19171 | 9.27434 | *GG* |
| Africans | 77 | YRI | Y071 | NA19140 | 8.29123 | *AG* |
| Africans | 78 | YRI | Y012 | NA18859 | 9.54905 | *AG* |
| Africans | 79 | YRI | Y060 | NA19119 | 8.64101 | *AG* |
| Africans | 80 | YRI | Y012 | NA18860 | 9.00049 | *AG* |
| Africans | 81 | YRI | Y051 | NA19207 | 8.92687 | *AG* |
